# A Novel TLR4 Inhibitor DB03476 Rescued Renal Inflammation in Acute Kidney Injury Model

**DOI:** 10.3390/ijms27010454

**Published:** 2025-12-31

**Authors:** Yi-Fan Zhang, Yu-Xuan Ma, Shi-Jie Wei, Bo Yang, Yun-Hua Ji, Zheng-Xiang Qi, Xin-Yu Shi, Long-Long Zhang, Xiao-Zheng Fan, Xiao-Jian Yang

**Affiliations:** 1 Department of Urology, Fourth Military Medical University, Xi’an 710032, China; 2 Department of Medical Genetics, School of Basic Medical Sciences, Southern Medical University, Guangzhou 510515, China

**Keywords:** drug screening, acute kidney injury, TLR4 inhibitor, kidney organoid

## Abstract

Acute kidney injury (AKI) is a critical clinical syndrome characterized by a rapid decline in renal function, frequently resulting from ischemia, nephrotoxicity, or sepsis. It represents a major global health burden due to its high morbidity and mortality and its strong association with progression to chronic kidney disease. In this study, we identified a novel small-molecule TLR4 inhibitor, DB03476, via structure-based virtual screening targeting the intracellular TIR domain of murine Tlr4. Molecular dynamics simulations confirmed that DB03476 stabilizes Tlr4 without altering its global conformation. In a murine ischemia–reperfusion-induced AKI model, DB03476 administration significantly attenuated renal inflammation, macrophage infiltration, and apoptosis and suppressed the TLR4/MyD88/NF-κB pathway. Moreover, DB03476 exhibited cross-species efficacy by binding conserved residues in human TLR4 with high affinity. Functional validation using human kidney organoids confirmed its protective effects against inflammatory challenge. These results demonstrate DB03476 as a promising therapeutic agent for AKI through selective inhibition of TLR4-mediated inflammatory responses.

## 1. Introduction

Acute kidney injury (AKI), characterized by an abrupt decline of kidney function, poses a global public health challenge with considerable mortality rates [1,2]. AKI encompasses a spectrum of etiologies from sepsis to nephrotoxin exposure. Among these, ischemia/reperfusion injury (IRI) is a predominant cause, particularly following cardiac surgery, renal transplantation, or hypovolemic shock [3,4,5]. The key pathophysiology of IRI-induced AKI is renal tubular cell injury and death, accompanied by peritubular endothelial dysfunction and inflammatory cell infiltration [5,6,7]. Despite its clinical significance, effective therapeutic strategies to promote renal recovery remain limited, underscoring an urgent need to elucidate novel molecular targets and therapeutic interventions [8].

Increasing evidence supports the significant role of pattern recognition receptors (PRRs) in cell death caused by AKI, such as Toll-like receptors (TLRs) [9]. Toll-like receptor 4 (Tlr4), a conserved core pattern recognition receptor in innate immunity, serves as a key model for studying mammalian immune responses. The 835-amino acid murine Tlr4 protein comprises an extracellular domain (ECD) with leucine-rich repeats (LRRs) for ligand binding (e.g., LPS/MD-2 complex), a transmembrane domain, and an intracellular Toll/IL-1 receptor (TIR) domain [10,11]. Ligand binding induces dimerization, enabling the TIR domain to recruit adaptors MyD88 and TRIF, initiating MyD88-dependent pro-inflammatory cytokine production (e.g., TNF-α, IL-6) and TRIF-dependent type I interferon responses [12,13,14]. Targeting the ECD with inhibitors like LPS antagonists faces limitations, including microenvironmental sensitivity and inability to block endogenous ligands (e.g., HMGB1), while full-length antibodies risk impairing immune surveillance. In contrast, the intracellular TIR domain acts as the essential convergence point for both signaling pathways.

Selective TIR inhibition offers a strategy to differentially suppress pathological pro-inflammatory cytokine storms without broadly compromising interferon pathways, potentially reducing secondary infection risks associated with immunosuppression.

Therefore, developing specific small-molecule inhibitors against the murine TLR4 TIR domain represents a promising therapeutic approach for sepsis, autoimmune disorders, and chronic inflammation. In this study, we identified a new small-molecule TLR4 inhibitor that could rescue AKI. Our data demonstrated the successful synthesis of the TLR4 inhibitor DB03476 and further explored its inhibitory effect on TLR4 protein expression in AKI model.

## 2. Results

### 2.1. Structural Modeling of Murine Tlr4 Intracellular Domain and Identification of Potent Inhibitors Targeting Cys745 via Virtual Screening and Molecular Dynamics Simulation

**Figure 1:**
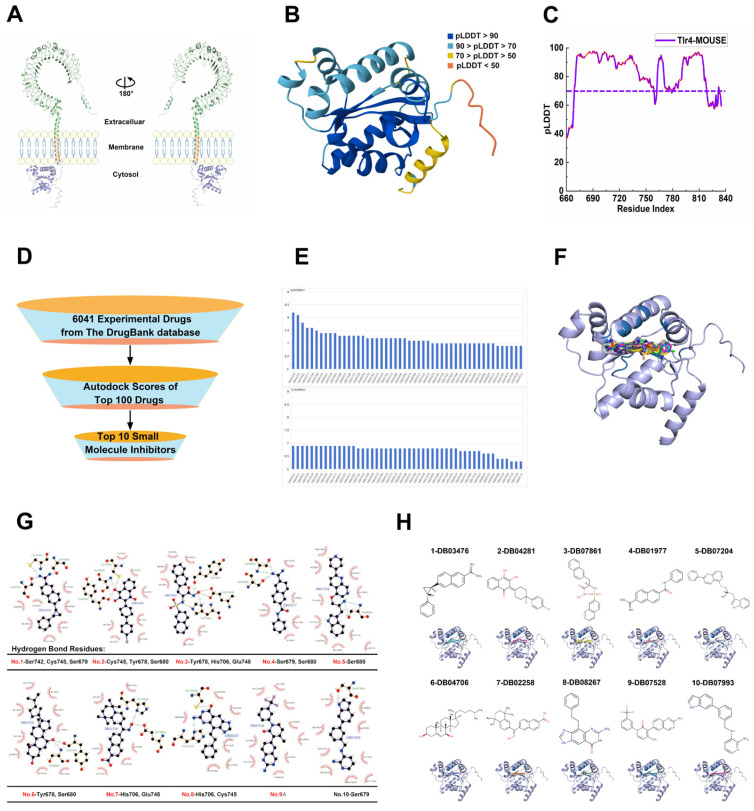
Structural modeling and virtual screening of the murine Tlr4 intracellular domain. (**A**) Tertiary structure of full-length murine Tlr4The model depicts the domain organization of the 835-amino acid mouse Tlr4 protein. Structural domains are color-coded: the N-terminal signal peptide (residues 1–25, light blue; cleaved in the mature protein), the extracellular domain (residues 26–638, light green) responsible for ligand recognition, the transmembrane helix (residues 639–659, orange), and the TIR domain (residues 660–835, purple) essential for intracellular signal transduction. (**B**) AlphaFold3-predicted structure of the Tlr4 intracellular domain. Colors represent local confidence (pLDDT): Very high (>90, dark blue), Confident (70–90, light blue), Low (50–70, yellow), Very low (<50, orange). (**C**) Per-residue pLDDT score distribution for the intracellular domain (residues 660–835). (**D**) Schematic workflow of the virtual screening campaign targeting the Tlr4 protein. (**E**) Top 100 small-molecule compounds identified by virtual screening against the murine Tlr4 protein, with corresponding binding energy scores. (**F**) Structural alignment of the top-10-ranked compounds docked within the putative ligand-binding pocket of murine Tlr4. (**G**) Two-dimensional ligand interaction diagrams of top-10-ranked compounds bound to murine Tlr4 + small molecules are represented as ball-and-stick models (purple). Hydrogen bond interactions are depicted as green dashed lines. Surrounding amino acid residues are shown as interaction diagrams (red). (**H**) Chemical structures and 3D binding modes of top-10-ranked compounds with murine Tlr4.

Drug simulation screening experiments were performed based on the structure model of TLR4 in mice (Figure 1A). Mutagenesis experiment studies have demonstrated that the inhibitor TAK-242 effectively suppresses TLR4 signaling by binding directly to the intracellular domain of TLR4, specifically targeting residue Cys747 in the human protein, corresponding to residue Cys745 within the murine Tlr4 intracellular segment studied here. We employed AlphaFold3 to model the structure of the murine Tlr4 intracellular domain to discover novel, potentially more potent inhibitors. The predicted conformation exhibited a high predicted TM-score (pTM) of 0.79, indicating a close approximation to the native protein structure (Figure 1B). Within the model, the structurally rigid core region displayed high confidence (pLDDT > 70), while regions with lower confidence (pLDDT < 70) were predominantly localized to peripheral areas distant from the putative ligand-binding pocket, supporting the overall reliability of the predicted conformation (Figure 1C). Subsequent virtual screening against the modeled Tlr4 protein utilized a library of 6041 experimentally validated bioactive small-molecules from the DrugBank database (Figure 1D). Analysis of the top 100 compounds ranked by binding affinity revealed that the ten highest-scoring molecules consistently occupied an identical binding site on the protein (Figure 1E,F). This conserved binding pocket is defined by the following amino acid residues: Phe675, Val676, Ile677, Tyr678, Ser679, Ser680, Gln681, His706, Phe710, Ile716, Ile720, Phe739, Ser742, Trp744, Cys745, Glu748, and Ile751.

Detailed analysis of molecular features and 2D binding environments reveals that high-affinity compounds predominantly contain aromatic ring systems, consistent with the hydrophobic character of the binding pocket (Phe675, Val676, Ile677, Phe710, Ile716, Ile720, Phe739, Ile751). Notably, an inverse correlation exists between binding energy scores and the number of polar interactions formed with residues including Ser742, Tyr678, Ser679, Ser680, His706, and Glu748. Three top-ranked compounds—DB03476 (No.1), DB04281 (No.2), and DB08267 (No.8)—directly engage the critical Cys745 residue via hydrogen bonding (Figure 1G), representing a potential mechanism for Tlr4 functional inhibition. These polar residues (Cys745, Ser742, Tyr678, Ser679, Ser680, His706, Glu748) constitute promising targets for enhancing inhibitor specificity and potency through rational polypharmacology approaches.

Further examination of 3D binding modes demonstrates that the most potent inhibitors (DB03476, DB04281, DB08267) share a common structural feature: a hydrophilic head group targeting Cys745 paired with an aromatic hydrophobic tail. This structural motif positions the hydrophobic moiety toward the pocket’s apolar residues to maximize van der Waals contacts, while the polar head maintains specific hydrogen bonding with Cys745 (Figure 1H).

Building upon our original analysis, we have further conducted a multi-dimensional evaluation and validation of the predicted structures. First, we performed Predicted Aligned Error (PAE) analysis on the five predicted conformations (model 0–model 4) of the murine Tlr4 intracellular domain generated by AlphaFold3. The results show high overall confidence for all models (Figure S1A–E), with only a few flexible loop regions exhibiting relatively higher PAE values (Figure S1F).

Building on this, we employed an ensemble docking strategy by performing molecular docking of the candidate compound DB03476, obtained from virtual screening, with each of the model 0–model 4 structures. The results revealed high structural consistency among model 0 to model 4, with RMSD values of 0.32, 0.29, 0.29, and 0.71 for models 1 to 4 relative to model 0, respectively. Furthermore, DB03476 stably bound near Cys745 across these models, with binding energy scores ranging from −7.4 to −8.2 kcal/mol, indicating favorable conformational compatibility with the pocket (Figure S2A–E). Notably, the binding mode of DB03476 in model 5 differed from the others, suggesting the possible existence of an alternative, potentially more stable binding pose that warrants further investigation through molecular dynamics simulations. Collectively, these supplemental analyses confirm that the selected model (model 0) exhibits high conformational consistency and reliability within the binding region, supporting the rationale for using this structure in subsequent virtual screening and mechanistic studies.

To clarify the potential advantages and mechanism of action of DB03476 compared to the known inhibitor TAK-242, we conducted a systematic comparative analysis. First, molecular docking revealed that TAK-242 could bind to both murine Tlr4 (binding energy: −6.0 kcal/mol) and human TLR4 (binding energy: −6.3 kcal/mol). However, its binding energies were higher than those of DB03476 with the corresponding proteins (−8.2 kcal/mol and −7.7 kcal/mol, respectively), suggesting that DB03476 possesses stronger potential binding affinity. Further docking studies using the constructed Cys745Ala (murine) mutant showed that the binding energy of TAK-242 to this mutant increased significantly to +24.5 kcal/mol, indicating that its action is highly dependent on covalent binding to Cys745. In contrast, DB03476 maintained a relatively low binding energy under the same mutant conditions, demonstrating that it also exerts stable inhibitory effects at the Cys745 site. This suggests that its mode of action may share common features with that of TAK-242 (Figure S3A–C).

In addition, we conducted 100 ns molecular dynamics simulations to evaluate the stability of the complexes. The results showed that the potential energy of the Tlr4–C745A–TAK-242 system (approximately −5.7 × 10^5^ kJ/mol) was significantly higher than that of the other systems (approximately −7.6 × 10^5^ kJ/mol). Moreover, its RMSD (approximately 8 Å) was considerably higher than that of the Tlr4–DB03476 (approximately 4 Å), Tlr4 (approximately 5.8 Å), and Tlr4–TAK-242 (approximately 5.6 Å) systems. These findings indicate that the binding of TAK-242 to the protein becomes highly unstable after the mutation, whereas DB03476 demonstrates superior conformational maintenance capability (Figure S4A–D). In summary, DB03476 exhibits better binding properties and stability than TAK-242 at the computational level. To clarify whether its action similarly depends on Cys745, we have included wet-lab validation in our subsequent research plan, which involves site-directed mutagenesis (Cys745/747Ala) combined with cell-based reporter gene assays. This will further elucidate its mechanism of action and translational potential.

### 2.2. Identification of High-Affinity Murine Tlr4 Inhibitors via Virtual Screening: Mechanistic Insights from Molecular Dynamics Simulation, PCA and Comparative Analysis with TAK-242

**Figure 2:**
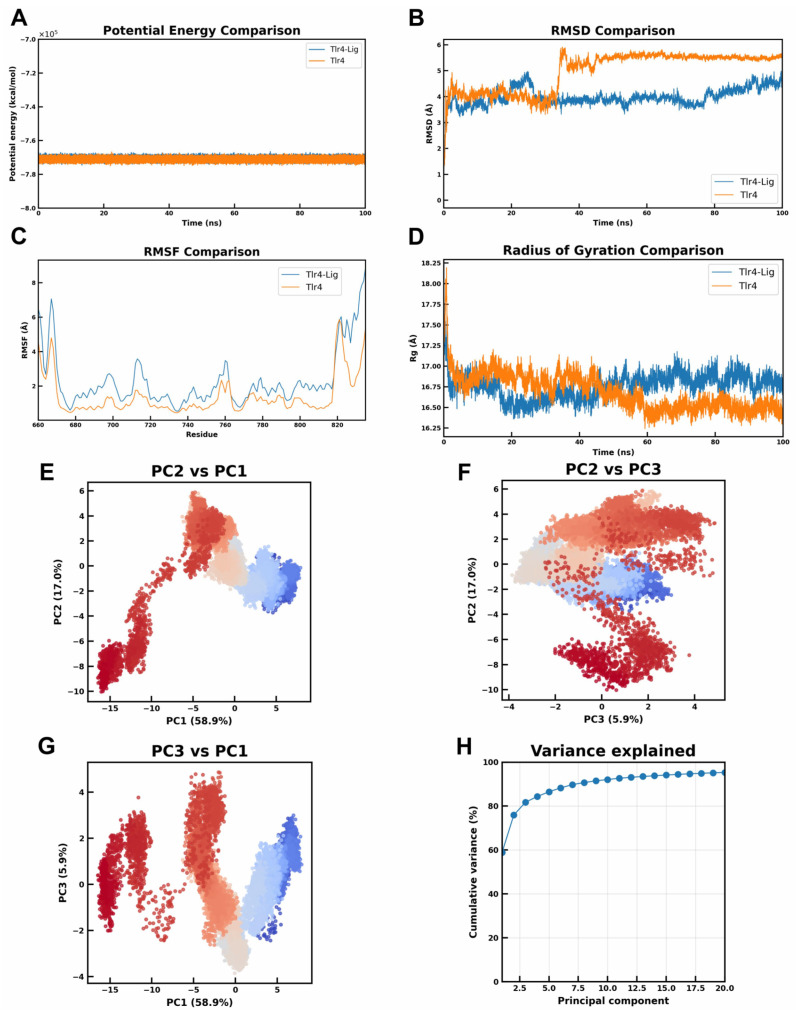
All-atom molecular dynamics simulations of murine Tlr4 and Tlr4-Lig systems and conformational landscape analysis of murine Tlr4-DB03476 complex. (**A**) Potential energy of the Tlr4 system over time with (blue) and without (orange) the inhibitor. Both systems reach similar stable energy levels, with the inhibitor-bound system achieving a slightly more negative potential energy, indicating a more stable state. (**B**) RMSD comparison between Tlr4 in the presence and absence of the small-molecule inhibitor. The Tlr4-inhibitor complex (blue) shows a lower and more stable RMSD than Tlr4 alone (orange), indicating enhanced overall structural stability with ligand binding. (**C**) RMSF per-residue profiles for Tlr4 with (blue) and without (orange) the inhibitor. Overall fluctuations are similar, but the inhibitor-bound Tlr4 shows slightly reduced flexibility at certain residues, suggesting a minor stabilizing effect on local regions. (**D**) Radius of gyration (Rg) of Tlr4 over time in the presence (blue) and absence (orange) of the inhibitor. The Rg stabilizes at comparable values in both simulations, with the ligand-bound Tlr4 initially slightly more compact. This indicates that the inhibitor does not significantly alter the overall size of Tlr4, preserving the protein’s global conformation. (**E**) Projection of molecular dynamics trajectories onto the PC2 vs. PC1 subspace. Early simulations (blue) transition toward late-stage conformations (red) along a well-defined pathway (PC1: 58.9%; PC2: 17.0%). (**F**) Trajectory distribution in PC2 vs. PC3 subspace (PC2: 17.0%; PC3: 5.9%). (**G**) Conformational sampling in PC3 vs. PC1 projection (PC1: 58.9%; PC3: 5.9%). (**H**) Variance contribution of principal components (cumulative variance: 81.8%).

We performed all-atom molecular dynamics simulations of Tlr4 with and without the DB03476 inhibitor to evaluate its impact on protein stability. The inhibitor-bound Tlr4 system shows a lower root-mean-square deviation (RMSD) than the apo form (Figure 2B), indicating less deviation from the initial structure and enhanced stability. The radius of gyration (Rg) remains similar between the two systems (Figure 2D), suggesting that the inhibitor does not induce any significant expansion or collapse of the protein and thus preserves Tlr4’s global conformation.

Residue-level flexibility (RMSF) is broadly comparable in both simulations, although certain regions of Tlr4 exhibit reduced fluctuations in the presence of the inhibitor (Figure 2C). This localized decrease in mobility implies that the ligand can stabilize specific segments of the protein. Additionally, the Tlr4-Lig system exhibits a slightly lower overall potential energy (Figure 2A), consistent with a more stable state. In summary, the inhibitor has a stabilizing effect on Tlr4, enhancing its structural stability without significantly altering the protein’s overall structure.

Principal component analysis (PCA) of the Tlr4-DB03476 complex trajectory demonstrated constrained conformational evolution. The system progressed along a narrow, well-defined transition pathway from early-stage (blue) to late-stage (red) conformers, culminating in tight clustering indicative of reduced conformational diversity and enhanced stability (Figure 2E–G). The first two principal components (PC1 and PC2) accounted for 76% of total variance, with the first three components collectively explaining 82% (Figure 2H). This high cumulative variance indicates that the system’s dominant fluctuations are confined to few collective modes. Critically, DB03476 binding imposes significant restrictions on TLR4’s accessible conformational landscape, guiding the protein along a specific transition pathway toward a stabilized bound state.

The results from molecular dynamics simulations show that for the Tlr4–DB03476 system, the RMSD before and after the 100 ns simulation was 1.41 Å, with DB03476 consistently bound near Cys745 and maintaining polar interactions with Tyr678. Notably, the β-carbon atom of the α,β-unsaturated amidine group (–CH=CH–C(=NH)–NH_2_) in DB03476 was in close proximity to the sulfur atom of Cys745, suggesting a potential covalent binding tendency (Figure S5A). In comparison, the Tlr4–TAK-242 system exhibited an RMSD of 1.04 Å, with TAK-242 maintaining stable interactions with Cys745 and Glu748 (Figure S5B). In contrast, for the mutant Tlr4–C745A–TAK-242 system, TAK-242 clearly deviated from its original binding site after simulation (Figure S5C), further confirming its high dependence on covalent modification of Cys745 for bindi.

### 2.3. Synthesis of Compound DB03476 Synthesis

**Figure 3:**
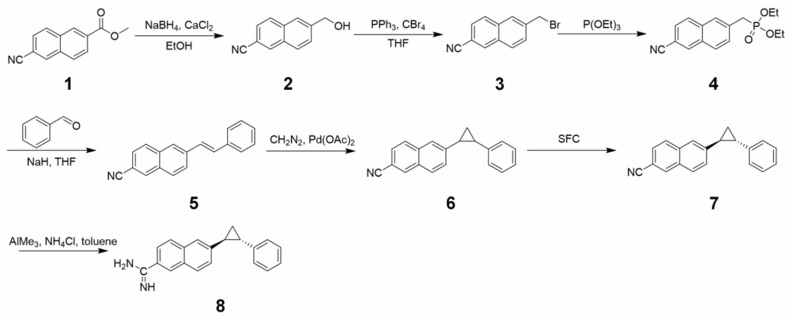
Synthetic pathway to DB03476. Methyl 6-cyano-2-naphthoate (**1**) was reduced with CaCl_2_/NaBH_4_ in THF/EtOH, worked up with H_2_O/KHSO_3_/HCl, DCM-extracted, and recrystallized to give 6-(hydroxymethyl)-2-naphthonitrile (**2**). 2 reacted with CBr4/PPh3 in THF, concentrated, flash CC-purified (50% DCM/hexanes) to afford 6-(bromomethyl)-2-naphthonitrile (**3**). 3 in triethyl phosphite was heated to 150 °C, precipitated with ether/hexanes to give (6-cyano-2-naphthyl) methylphosphonate (**4**). 4 was deprotonated by NaH, reacted with benzaldehyde, extracted, CC-purified to give diethyl (E)-6-styryl-2-naphthonitrile (**5**). 5 with diazomethane/Pd(OAc)2 gave 6-(2-phenylcyclopropyl)-2-naphthonitrile (**6**). 6 was SFC-purified to 6-((1S,2S)-2-phenylcyclopropyl)-2-naphthonitrile (**7**). 7 with AlMe_3_/NH_4_Cl in toluene, worked up with HCl, and Prep-HPLC-purified to yield 6-((1S,2S)-2-phenylcyclopropyl)-2-naphthimidamide (**8**).

The synthesis procedure is depicted in Figure 3. A solution of methyl 6-cyano-2-naphthoate (**1**) (1.0 eq) in THF at room temperature was treated with a solution of calcium chloride (1.5 eq) in ethanol and sodium borohydride (1.5 eq). The mixture was stirred for 16 h, treated sequentially with water, 10% KHSO_3_, and 1M HCl, and extracted with dichloromethane. The combined extracts were washed with water and brine, dried (MgSO_4_), filtered, and concentrated. The concentrate was recrystallized from ether/hexanes to afford the desired product 6-(hydroxymethyl)-2-naphthonitrile (**2**). A solution of 2 (1.0 eq) in THE (80 mL) at room temperature was treated with carbon tetrabromide (1.5 eq) and triphenylphosphine (1.5 eq), stirred for 16 h, and concentrated. The concentrate was purified by flash column chromatography on silica gel with 50% dichloromethane/hexanes to afford the desired product 6-(bromomethyl)-2-naphthonitrile (**3**). A suspension of 3 (1.0 eq) in triethylphosphite (10.0 mL) was heated to 150 °C, stirred for 3 h, cooled to room temperature, and treated with diethyl ether and hexanes. The resulting precipitate was collected by filtration and dried to afford the desired product diethyl (6-cyano-2-naphthyl) methylphosphonate (**4**). A solution of 4 (1.0 eq) in THF at 0 °C was treated with NaH (1.5 eq), stirred for 30 min, treated with benzaldehyde (1.2 eq), and warmed to room temperature over 2 h. The mixture was quenched with water and extracted with dichloromethane. The combined extracts were washed with water and brine, dried (MgSO_4_), filtered, and concentrated. The concentrate was purified by flash column chromatography on silica gel with dichloromethane to afford the desired product diethyl (E)-6-styryl-2-naphthonitrile (**5**). A solution of 5 (1.0 eq) in THF (10 mL) was added dropwise to diazomethane at 0 °C, treated with Pd (OAc)2, stirred for 20 min, filtered, and concentrated to provide the desired product. The reaction mixture was concentrated on silica gel and purified by silica gel chromatography to give the desired compound 6-(2-phenylcyclopropyl)-2-naphthonitrile (**6**), which was then purified by SFC to give the product 6-((1S,2S)-2-phenylcyclopropyl)-2-naphthonitrile (**7**). A solution of 7 (1 eq), NH4Cl in toluene was added to AlMe3 at 0 °C, then the reaction was stirred for 6 h at 100 °C, treated with 10% HCl (10 mL), stirred for 24 h at room temperature, concentrated, and purified by medium pressure liquid chromatography Prep-HPLC (TFA condition) to provide the title compound (**8**).

### 2.4. DB03476 Could Alleviate Kidney Inflammation Under AKI Model

**Figure 4:**
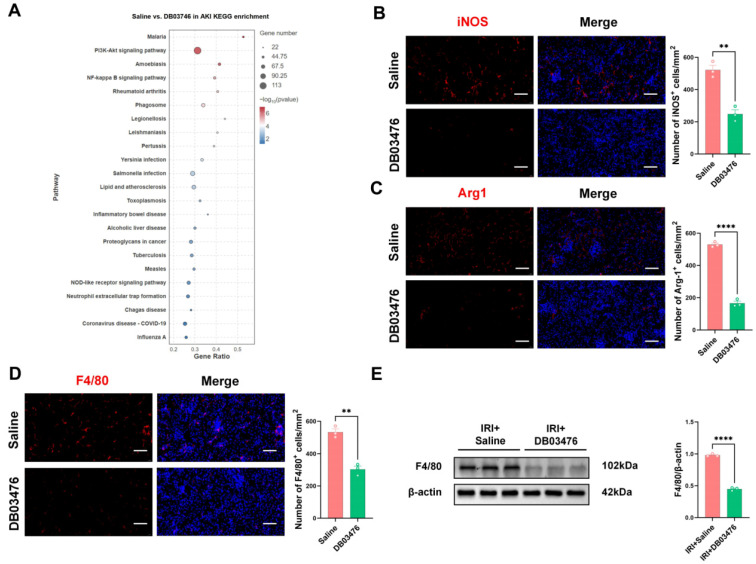
DB03476 inhibits Tlr4 pathway enrichment and macrophage infiltration/polarization in the kidneys of AKI mice. (**A**) RNA-seq analysis showed significant differences in multiple TLR4-enriched signaling pathways between the saline group and DB03476-treated group in renal tissues of the AKI model. (**B**,**C**) Immunofluorescence staining for iNOS and Arg1 in drug-treated renal tissues. (**D**) Immunofluorescence staining for F4/80 in drug-treated renal tissues. Bars = 80 μm. (**E**) The protein level of F4/80 in drug-treated renal tissues was detected by Western blotting. The sample size was n = 3 per group. Data are presented as mean ± SEM. ** *p* < 0.01, **** *p* < 0.0001.

**Figure 5:**
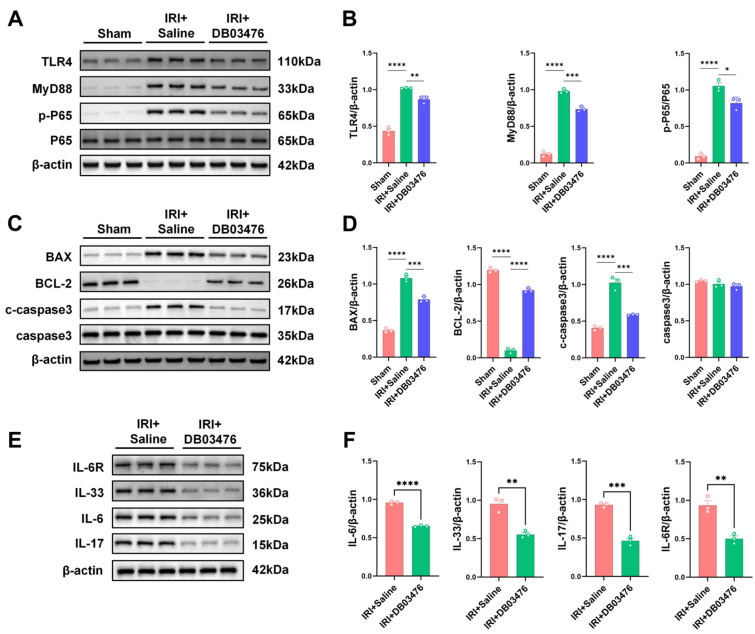
DB03476 suppresses TLR4/MyD88/NF-κB signaling pathway activation, renal cell apoptosis, and inflammatory factor expression in AKI mice. (**A**,**B**) Renal p-P65, MyD88,TLR4, and P65 expression. (**C**,**D**) c-caspase3, Bcl-2, Bax, and caspase-3 protein levels in the kidney of mice were quantified using Western blotting. (**E**,**F**) IL-6, IL-6R, IL-17, and IL-33 protein expressions were measured using Western blotting. The sample size was n = 3 per group. Data are presented as mean ± SEM. * *p* < 0.05, ** *p* < 0.01, *** *p* < 0.001, **** *p* < 0.0001.

To verify the inhibitory efficiency of DB03476 on the expression of TLR4 protein, we added LPS to the cultured macrophages in vitro to induce their polarization. We found that after the addition of DB03476, the expression level of TLR4 in macrophages significantly decreased (Figure S6A,B), and it effectively inhibited the polarization of M1 and M2 (Figure S6C,D). Further, we conducted in vivo experiments in mice kidney under the AKI model to verify the results. Firstly, we confirmed through HE staining of the heart, liver, spleen, and lung that there was no biological toxicity after the injection of DB03476 (Figure S7). We conducted RNA-seq analysis on the kidneys of the saline group after the AKI model and the DB03476 group. The results showed that a large number of pathways enriched with Tlr4 exhibited statistically significant differences (Figure 4A). The expression of F4/80 in the kidneys by DB03476 administration was significantly reduced (Figure 4B,C), and the polarization of macrophages M1 and M2 was also significantly decreased (Figure 4D,E), also including CD86 and CD206 (Figure S8A,B). A solid piece of evidence demonstrated that DB03476 had a significant inhibitory effect on the TLR4/MyD88/NF-κB signaling pathway. The quantitative results of Western blotting showed that the expression levels of TLR4, MyD88, and p65 were significantly decreased (Figure 5A,B). Furthermore, we found that DB03476 did not cause any significant changes in the expression levels of TLR2, TLR7, and TLR8 (Figure S8C–E). The pre-treatment with DB03476 could effectively alleviate the apoptosis of renal cells caused by AKI, and the protein expressions of Bax, Bcl-2, and cleaved caspase3 show a significant downward trend (Figure 5C,D). The inflammatory factors associated with acute kidney injury, such as IL-6, IL-6R, IL-17, and IL-33, also showed significantly decreased expression levels by DB03476 administration (Figure 5E,F).

### 2.5. DB03476’s Structural Basis for Inhibiting the Intracellular Domain of TLR4 in Mice and Human

**Figure 6:**
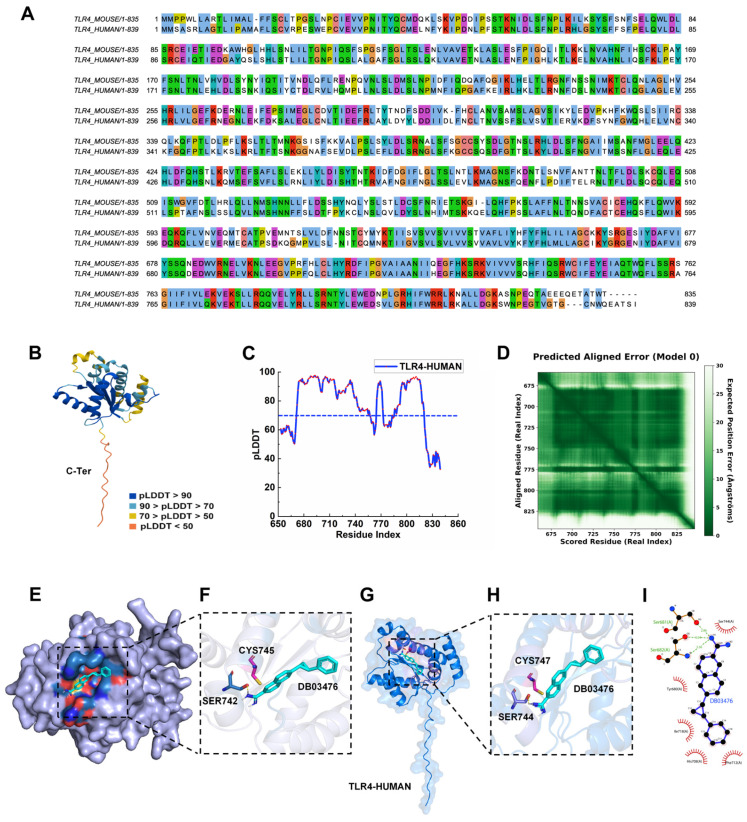
Structural modeling of human TLR4 and homologous sequence alignment and structural basis of DB03476 inhibition in murine and human TLR4 intracellular domains. (**A**) AlphaFold3-predicted structure of the human TLR4 intracellular domain. Color gradient indicates local confidence (pLDDT): dark blue (highest) to orange (lowest). (**B**) Per-residue pLDDT profile for the intracellular domain (residues 653–839). (**C**) Clustal alignment of full-length human and murine TLR4 protein sequences. (**D**) Aligned Error (PAE) plots for the Alphafold3 predicted model 0 of the TLR4 intracellular domain. The color gradient (dark green to white) represents PAE values from 0 to 30. The horizontal and vertical axes correspond to the aligned residue indices (real index). (**E**) Overall binding pose of DB03476 (light blue sticks) within the murine Tlr4 binding pocket (protein surface: purple; key residues: deep blue sticks). (**F**) Detailed interaction map of DB03476 with murine Tlr4 (cartoon mode). Key functional residue Cys745 (pink sticks) and polar interaction partners (deep blue sticks) are shown. Hydrogen bonds indicated by yellow dashed lines. (**G**) Global binding mode of DB03476 in human TLR4 (protein cartoon: blue; binding pocket residues: purple sticks). (**H**) Molecular interactions between DB03476 and human TLR4. Catalytic residue Cys747 (pink sticks) and polar contact residues (purple sticks) highlighted. (**I**) 2D ligand interaction diagram of DB03476 with human TLR4 residues. Hydrogen bonds (green dashes) and surrounding residues (red spikes) were indicated.

Sequence alignment revealed 66% identity between human and murine TLR4 protein (Figure 6A). To evaluate the translational potential of our virtual screening hits, we modeled the human TLR4 intracellular domain using AlphaFold3 (version 3.0.0). The predicted structure achieved a high pTM score of 0.75, indicating close approximation to the native conformation (Figure 6B). Crucially, all residues within the ligand-binding pocket exhibited pLDDT values > 70 (Figure 6C), demonstrating exceptional local confidence in this functionally critical region. These robust structural models provide a reliable foundation for subsequent experimental validation and rational inhibitor design targeting human TLR4. Additionally, we have supplemented the main text with PAE analysis for the human TLR4 protein (Figure 6D).

Following structural validation of human TLR4, molecular docking was performed with the top-ranked compound DB03476 and compared with its murine Tlr4 binding mode. Both systems exhibited high conformational complementarity between DB03476 and their respective binding pockets (Figure 6E,G), with conserved polar interactions observed at Ser742 (murine) and its structural homolog Ser744 (human) (Figure 6F,H). DB03476 demonstrated strong binding affinity to human TLR4 (ΔG = −7.7 kcal/mol), forming additional hydrogen bonds with Ser681 and Ser682 (Figure 6I). Critically, the inhibitor occupies the catalytic pocket through steric blockade of the functionally essential cysteine residues (Cys745 in murine/Cys747 in human), concurrently maintaining favorable interactions with both orthologs. This conserved binding mechanism and dual-species efficacy establish DB03476 as a high-potency TLR4 inhibitor candidate with translational potential.

### 2.6. Constructing Kidney Organoids to Verify the Inhibitory Effect of DB03476 on Human TLR4

**Figure 7:**
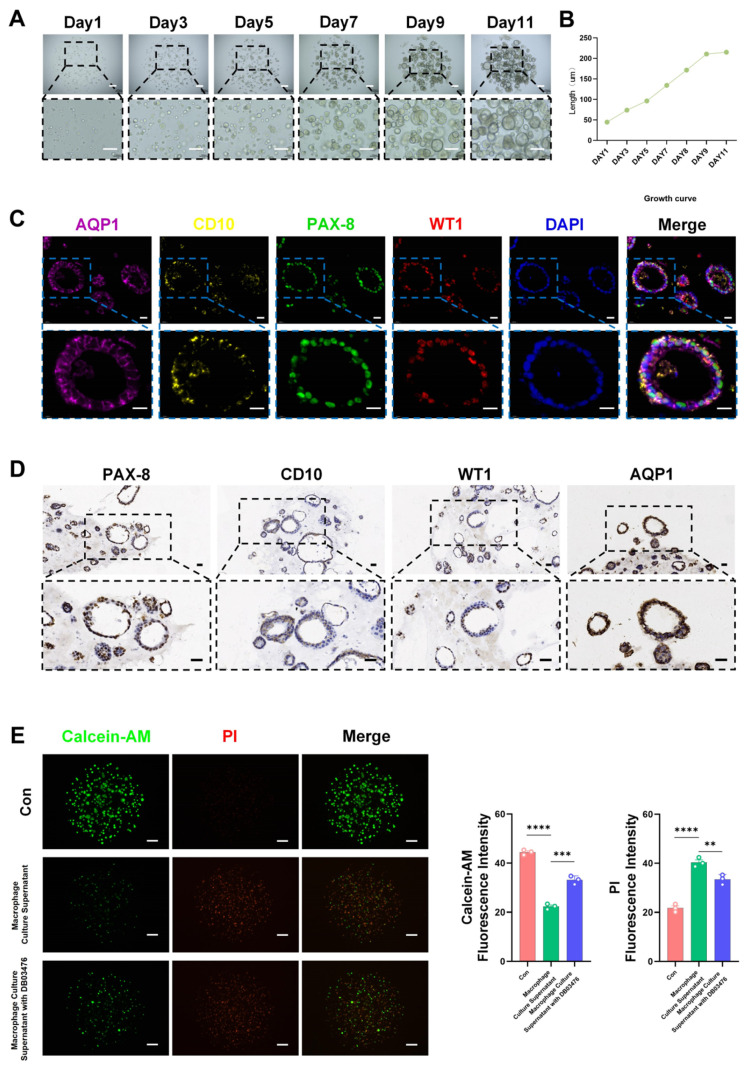
Inhibition of human TLR4 by DB03476 verified in kidney organoid model. (**A**,**B**) Schematic of kidney organoid cultivation process showing successful establishment of 3D organoid model after 11 days of culture. Growth curve showing progressive increase in organoid diameter with culture duration. Bars = 200 μm. (**C**,**D**) Immunofluorescence and immunohistochemistry staining for renal markers, including AQP1 (purple), CD10 (yellow), PAX-8 (green) and WT1 (red), confirmed the functional maturation of organoids. Bars = 0.02 mm (**E**) Live/dead cell viability assay using Calcein-AM (viable cells, green) and PI (dead cells, red) staining. Bars = 200 μm. The sample size was n = 3 per group. Data are presented as mean ± SEM. ** *p* < 0.01, *** *p* < 0.001, **** *p* < 0.0001.

In order to simulate the effect of DB03476 in human kidneys, we constructed a kidney organoid model. We cultivated the kidney organoids model for 11 days (Figure 7A). The growth curve showed that the diameter of the organoids increased as the number of culture days increased (Figure 7B). The immunofluorescence results of the biological markers AQP1, CD10, PAX-8, and WT1 in the kidneys showed that the organoid model was matured (Figure 7C), and the immunohistochemistry also reflected similar results (Figure 7D). Since the organoid model cannot replicate the functions of macrophages in human kidneys, we added the culture supernatant of macrophages cultured with LPS to the organoids and then examined the survival status of the organoid cells. We used Calcein-AM and PI dyes to mark the survival and death of cells. The results showed that the group with supernatant from DB03476-treated macrophages had significantly more surviving cells and significantly fewer dead cells in the organoid samples (Figure 7E).

## 3. Discussion

In this study, we screened a novel small-molecule inhibitor of TLR4, DB03476, from the DrugBank database. We demonstrated the inhibitory effect of DB03476 on TLR4-mediated macrophage infiltration and the downstream signaling cascade in AKI models. We also verified the inhibitory effect of DB03476 on macrophage infiltration using a humanized kidney organoid model.

For a long time, TLR4 has been the focus of attention as an important receptor for pathological processes such as cell death and inflammatory responses [15,16,17]. The development of effective and stable targeted inhibitors for TLR4 has long been regarded as a key issue in the related field [18,19,20]. In previous studies, it was reported that the TLR4 inhibitors caused problems such as protein swelling or collapse, which led to the disruption of the overall structure of the TLR4 protein [21,22]. The novel TLR4 inhibitor we developed has been verified through molecular dynamics simulation to be able to occupy the binding sites while maintaining the stable structure of the TLR4 protein. The new TLR4 inhibitor we developed has been verified through molecular dynamics simulation to be able to occupy the binding sites while maintaining the stable structure of the TLR4 protein. And, without causing any toxic effects, it effectively inhibited the kidney inflammation in the mouse AKI model. The species-specific sequence differences between humans and mice have always been the fundamental reason why many small-molecule compounds have failed to reach clinical application [23,24]. We simulated the physiological conditions of the human kidney using kidney organoids, and DB03476 also exhibited excellent inhibitory effects.

The most significant contribution of this study lies in the development of a novel TLR4 inhibitor, which was used to rescue acute kidney injury in the AKI model. However, TLR4 is involved in multiple pathological pathways. We have not been able to study other cellular processes apart from the inflammatory pathways mediated by TLR4. This has become a limitation of this research. Furthermore, we did not compare the inhibitory efficiency with any other reported TLR4 inhibitors.

## 4. Materials and Methods

### 4.1. Animals

C57BL/6J mice were sourced from the animal facility of the Air Force Medical University. All mice were maintained in specific pathogen-free conditions and housed under a 12 light/12 dark cycle at a controlled temperature (22–24 °C), with free access to water and standard rodent chaw. All animal experiments were carried out according to the protocols and animal ethics approved by the Animal Care and Use Committees of Air Force Medical University. This experiment adhered to the ARRIVE guidelines.

### 4.2. Macrophage Culture

RAW264.7 cells, a mouse leukemic monocyte/macrophage cell line, were purchased from the American Type Culture Collection (ATCC, Manassas, VA, USA) and maintained in DMEM supplemented with 10% heat-inactivated FBS, 100 U/mL penicillin, and 100 μg/mL streptomycin under a humidified atmosphere of 5% CO_2_ at 37 °C.

### 4.3. Protein Structure Modeling Using AlphaFold3

The tertiary structures of human Toll-like Receptor 4 (TLR4) (Homo sapiens) and mouse Tlr4 (Mus musculus) were predicted de novo using AlphaFold3 (version 3.0.0). Canonical protein sequences for TLR4 (UniProt accession O00206 for human; Q9QUK6 for mouse) were retrieved from the UniProt Knowledgebase. Structure predictions were executed via the local AlphaFold3 API. No templates were explicitly provided, leveraging AlphaFold3’s end-to-end deep learning architecture to generate atomic coordinates ab initio from the primary amino acid sequence [25].

For each target, five initial models were generated per run. The model with the highest predicted confidence (pLDDT > 70 for >90% of residues in the core domain) was selected as the representative structure. Models were visualized and analyzed using PyMOL Molecular Graphics System (v3.0.3, Schrödinger LLC, New York, NY, USA) (https://pymol.org/2/) (accessed on 23 December 2025). Structural validation was performed using the AlphaFold3 output metrics (pLDDT per residue, Predicted Aligned Error), with regions of low confidence (pLDDT < 70) noted in subsequent analyses.

### 4.4. Virtual Screening Targeting Murine Tlr4 Intracellular Domain

The tertiary structure of the Tlr4 intracellular segment (residues 660–835) was predicted de novo using AlphaFold3. The model exhibited high confidence. The predicted structure was prepared by adding polar hydrogens and assigning Kollman charges using AutoDock Tools 1.5.7. A library of 6041 experimentally validated small-molecule drugs from DrugBank (Experimental Drug subset, Version 5.1.7) was energy-minimized with the UFF force field in Open Babel 3.1.1 and converted to PDBQT format. A grid box centered at the functional residue Cys745 was defined with coordinates: Center: X = −12 Å, Y = 5.5 Å, Z = 2 Å; Dimensions: X = 11 Å, Y = 12 Å, Z = 15 Å. Virtual screening was executed using AutoDock Vina 1.2.3 (exhaustiveness = 32). Compounds were ranked by docking affinity (kcal/mol), and the top 100 hits were retained. Final top 10 inhibitors were selected based on binding energy and structural clustering analysis.

### 4.5. Homology-Based Sequence Alignment of TLR4

Multiple sequence alignment of human (UniProt: O00206) and murine (UniProt: Q9QUK6) TLR4 proteins was performed using Jalview 2.11.3 software. Canonical protein sequences were retrieved from UniProtKB and imported directly into Jalview. Alignment was executed with the Clustal Omega algorithm (version 1.2.4) under default parameters. Conservation analysis was conducted using the clustalX color scheme to visualize residue physicochemical properties, with conservation scores calculated via the Taylor entropy method.

### 4.6. Molecular Docking of DB03476 to Human TLR4 Intracellular Domain

High-precision docking was performed to characterize the binding mode of inhibitor DB03476 (DrugBank ID) to the human TLR4 intracellular domain (UniProt: O00206). The tertiary structure of TLR4 intracellular segment (residues 653–839) was predicted using AlphaFold3 with high confidence. The receptor structure was prepared in AutoDock Tools 1.5.7: polar hydrogens were added, Gasteiger charges assigned, and non-polar hydrogens merged. The ligand DB03476 was retrieved in 3D SDF format from DrugBank (version 5.1.9). Conformational optimization was executed using Open Babel 3.1.1 with the MMFF94 force field, followed by conversion to PDBQT format. Rotatable bonds were defined automatically. A grid box centered on the functional site Cys747 was constructed with dimensions 15 × 15 × 15 Å to ensure comprehensive sampling. Docking simulations used AutoDock Vina 1.2.3 with exhaustiveness = 32. The dominant conformation was selected based on binding affinity and structural consistency with the characteristic binding patterns of the TIR domain.

### 4.7. Molecular Dynamics Simulations of Tlr4 Systems

All-atom molecular dynamics (MD) simulations were performed for murine Tlr4 intracellular domain in two states: apo form and DB03476-bound complex. Initial structures were derived from the AlphaFold3-predicted model. The inhibitor DB03476 was parameterized using AMBER03 force field with GAFF2 topology generated via ACPYPE. Both systems were solvated in SPC water within a dodecahedral box (minimum 1.0 nm protein-wall distance), with Na^+^/Cl^−^ ions added to neutralize the charge. Energy minimization employed the steepest descent algorithm (convergence: 100 kJ·mol^−1^·nm^−1^; step size: 0.005 nm). Systems were equilibrated under NPT ensemble (300 K, 1 bar) for 400 ps. Protein backbone atoms were restrained (5000 kJ·mol^−1^·nm^−2^) during equilibration. Production simulations (120 ns/system) used a 1-fs integration time step with PME electrostatics (real-space cutoff: 1.2 nm) and van der Waals cutoff (1.2 nm) with energy/pressure dispersion correction.

Trajectory analysis included potential energy evolution to validate stability, backbone RMSD relative to initial structure, per-residue RMSF of Cα atoms, radius of gyration (Rg) for global compactness, principal component analysis (PCA) on Cα covariance matrix (GROMACS gmx covar/covar), and conformational clustering (GROMOS algorithm, cutoff 0.35 nm). All simulations used GROMACS 2024.1, with analyses performed on the last 100 ns of production trajectories.

### 4.8. RNA Sequencing and Bioinformatic Analysis

Total RNA was extracted using the Trizol reagent kit (Invitrogen, Carlsbad, CA, USA) according to the manufacturer’s protocol. RNA quality was assessed on an Agilent 2100 Bioanalyzer (Agilent Technologies, Palo Alto, CA, USA) and checked using RNase free agarose gel electrophoresis. After total RNA was extracted, eukaryotic mRNA was enriched by Oligo(dT) beads. Then, the enriched mRNA was fragmented into short fragments using fragmentation buffer and reverse-transcripted into cDNA with random primers. Second-strand cDNA were synthesized by DNA polymerase I, RNase H, dNTP, and buffer. Then, the cDNA fragments were purified with the QiaQuick PCR extraction kit (Qiagen, Venlo, The Netherlands), end-repaired, poly(A)-added, and ligated to Illumina sequencing adapters. The ligation products were size-selected by agarose gel electrophoresis, PCR-amplified, and sequenced using Illumina Novaseq 6000 by Omicsmaster Biotechnology Co., Ltd. (Guangzhou, China). When log2FC is greater than 1 and padj is less than 0.05, the significantly differentially expressed genes are selected. Bioinformatic analysis was performed using Omicsmaster, a real-time interactive online platform for data analysis (https://report.omicsmaster.com/) (accessed on 3 July 2025).

### 4.9. IRI Model and Compound Administration

Mice were randomly separated into three groups (n = 6/group): Sham, IRI + Saline, and IRI + DB03476. The IRI model was established as previously described [26]. A midline abdominal incision was made to expose both renal arteries, which were clamped with non-invasive vascular clips for 45 min. The clamps were removed to restore blood flow. Kidney tissue was collected 72 h post-surgery. Sham group underwent surgical exposure of the kidney without ischemia induction. In the IRI + Saline group, renal ischemia–reperfusion was induced by clamping the bilateral renal arteries for 45 min, followed by intraperitoneal injection of normal saline administered consecutively for 3 days. In the IRI + DB03476 group, treatment with DB03476 (50 mg/kg/day) was initiated immediately after the IRI procedure and continued for 3 consecutive days.

All mice were anesthetized with 1% (*w*/*v*) pentobarbital sodium solution, administered at a dose of 50 mg/kg via intraperitoneal injection before surgery. At the end of the experiments, all mice were euthanized by carbon dioxide via a gas anesthesia machine.

### 4.10. Immunofluorescence

For immunofluorescent staining, animals were sacrificed and perfusion fixed. Frozen sections (10–20 μm in thickness) were prepared. After blocking, primary antibodies were incubated overnight as follows: rabbit anti-iNOS antibody (1:150, ab178945, Abcam, Cambridge, UK, RRID:AB_2861417), mouse anti-Arginase-1 antibody (1:150, 66129-1-IG, Proteintech, Wuhan, China, RRID:AB_2881528), rat anti-F4/80 antibody (1:200, ab6640, Abcam, Cambridge, UK, RRID:AB_1140040), and mouse anti-TLR4 antibody (1:200, ab22048, Abcam, Cambridge, UK, RRID:AB_446735). After washing, sections were incubated with secondary antibodies conjugated with Alexa Fluor 594 (donkey anti-rabbit 711-585-152 or anti-mouse 715-585-151 IgG, 1:800, Molecular Probes, Eugene, OR, USA) or Alexa Fluor 488 (donkey anti-goat IgG, 705-545-147; anti-rat IgG, 712-545-153; or anti-rabbit IgG, 711-545-152, Molecular Probes, Eugene, OR, USA) for 2–4 h at room temperature. The nuclei were stained by DAPI (1:2000, Sigma D9542, St. Louis, MO, USA).

In the organoid staining part of this experiment, the following antibodies were used: rabbit anti-Aquaporin 1 antibody (1:100, 69343S, CST, Danvers, MA, USA); rabbit anti-WT1 antibody (1:100, 83535S, CST, Danvers, MA, USA); rabbit anti-CD10 antibody (1:500, 65534S, CST, Danvers, MA, USA); and rabbit anti-PAX8 antibody (1:2000, ab239363, Abcam, Cambridge, UK, RRID:AB_3665792). Co-staining was conducted using the five-color multiplex fluorescent staining kit (RC0086-45RM, RecordBio, Shanghai, China).

### 4.11. Western Blotting

For Western blotting, tissues were homogenized in RIPA buffer. Protein concentration was measured by the BCA assay. Proteins were separated by SDS-PAGE and transferred to the PVDF membrane. After blocking, membranes were incubated with rabbit anti-F4/80 antibody (1:1000, ab300421, Abcam, Cambridge, UK, RRID:AB_2936298), mouse anti-TLR4 antibody (1:1000, ab8376, Abcam, Cambridge, UK, RRID:AB_306522), rabbit anti-MyD88 antibody (1:1000, ab133739, Abcam, Cambridge, UK, RRID:AB_2637005), mouse anti-pp65 antibody (1:1000, ab53489, Abcam, Cambridge, UK, RRID:AB_879784), rabbit anti-p65 antibody (1:1000, ab32536, Abcam, Cambridge, UK, RRID:AB_776751), rabbit anti-Bax antibody (1:1000, ab32503, Abcam, Cambridge, UK, RRID:AB_725631), rabbit anti-Bcl-2 antibody (1:1000, ab182858, Abcam, Cambridge, UK, RRID:AB_2715467), rabbit anti-Caspase-3 antibody (1:1000, ab32351, Abcam, Cambridge, UK, RRID:AB_725946), rabbit anti-Cleaved Caspase-3 antibody (1:1000, ab32042, Abcam, Cambridge, UK, RRID:AB_725947), rat anti-IL-6R antibody (1:1000, ab83053, Abcam, Cambridge, UK, RRID:AB_1860516), rabbit anti-IL-33 antibody (1:1000, ab187060, Abcam, Cambridge, UK, RRID:AB_2894704), rabbit anti-IL-6 antibody (1:1000, ab233706, Abcam, Cambridge, UK, RRID:AB_2889391), and mouse anti-IL-17 antibody (1:1000, 66148-1-Ig, Proteintech, Wuhan, China, RRID:AB_2881544) overnight at 4 °C. Then, membranes were incubated with HRP-conjugated anti-rabbit, anti-mouse, or anti-rat (1:5000; SA00001-1, SA00001-2, SA00001-15, Proteintech, Wuhan, China, RRID:AB_2722565, RRID:AB_2722565, RRID:AB_2864369) for 1 h at room temperature. Bands were visualized with an ECL kit (Cat#:32106, Thermo, Waltham, MA, USA).

### 4.12. Organoid Construction and Processing and Subculture Steps

Tissues were rinsed 3 times with Hank’s Balanced Salt Solution (HBSS) containing antibiotics and then minced into 1 mm^3^ pieces using sterile scissors. Digestive solution was added, and the tissues were digested in a shaker at 37 °C for 0.5–1 h. Formulation of digestive solution was as follows: 0.001% DNase (Sigma-Aldrich, MO, USA), 1 mg/mL collagenase (Roche, IN, USA), 200 U/mL penicillin, 200 mg/mL streptomycin, and 0.5 mg/mL amphotericin B (2% antibiotics in total, Sigma) were added to Advanced DMEM/F12. After filtration through a 70 μm cell strainer, the filtrate was centrifuged at 300× *g* for 5 min at 4 °C. The supernatant was discarded, and 1 mL of red blood cell (RBC) lysis buffer was added to the pellet. The mixture was incubated on ice for 1 min to lyse RBCs. Subsequently, 2 mL of DMEM/F12 containing 2% penicillin-streptomycin was added to terminate the lysis reaction, followed by centrifugation at 300× *g* for 5 min at 4 °C. The supernatant was removed, and the cell pellet was resuspended in 100 μL of organoid medium. Cell counting was performed immediately after resuspension. Based on the cell counting results, Matrigel was added to resuspend and mix the cells thoroughly on an ice bath, adjusting the cell density to 10,000 cells/μL. The cell–Matrigel mixture was dispensed into 6-well plates at a volume of 10 μL per drop (60 μL total per well). The 6-well plate was then placed in a 37 °C incubator and inverted for 10 min to allow Matrigel solidification. Finally, 3 mL of organoid medium was added to each well of the 6-well plate, and subsequent organoid culture was carried out.

Pre-chilled PBS was used to aspirate and wash off the organoid Matrigel droplets. The collected droplets were centrifuged at 300× *g* for 5 min at 4 °C. The pellet was resuspended in PBS, followed by centrifugation at 250× RCF for 15 min at 4 °C. The supernatant was discarded. Then, 2 mL of Tryple Express (Invitrogen, Waltham, MA, USA) was added to the pellet and mixed thoroughly. The mixture was incubated at 37 °C for 10 min. Subsequently, 10 mL of DMEM/F12 supplemented with 10% fetal bovine serum (FBS), and 2% penicillin-streptomycin (P/S) was added, and the sample was centrifuged at 300× *g* for 5 min at 4 °C. The supernatant was removed. The pellet was resuspended in PBS and centrifuged at 300× *g* for 5 min at 4 °C. The supernatant was discarded. Organoid medium and Matrigel were mixed at a ratio of 1:5 (*v*/*v*), and the mixture was inoculated onto plates on an ice bath. This ice-bath operation was performed to prevent Matrigel from solidifying and denaturing due to temperature elevation. The plates were placed in an incubator for 10 min to allow Matrigel solidification. After that, organoid medium was added to each well for subsequent culture.

### 4.13. Statistical Analysis

All the analysis was made by experienced researchers who were blind to the experimental design. Data were expressed as mean ± SEM and analyzed by using GraphPad Prism 8.0 and SPSS 21.0 software. Two-tailed unpaired *t*-test and one-way ANOVA were adopted. The normality of data distribution was evaluated by the Shapiro–Wilk test. Statistical significance was assessed at levels of *p* < 0.05.

## Data Availability

The data presented in this study are not publicly available due to privacy concerns, but are available on request from the corresponding author.

## Supplementary Materials

The following supporting information can be downloaded at https://www.mdpi.com/article/10.3390/ijms27010454/s1.
