# Supplementary material for: A Novel TLR4 Inhibitor DB03476 Rescued Renal Inflammation in Acute Kidney Injury Model
Source: Int J Mol Sci. 2025 Dec 31;27(1):454. doi: 10.3390/ijms27010454 (PMC12787066; doi:10.3390/ijms27010454)
Supplement: Supplementary file 1 [file ijms-27-00454-s001.zip › Supplementary Information.pdf]

A novel TLR4 inhibitor DB03476 rescued renal inflammation in acute kidney injury model.

Yi-Fan Zhang [1,†], Yu-Xuan Ma [1,†], Shi-Jie Wei [2], Bo Yang [1], Yun-Hua Ji [1], Zheng-Xiang Qi [1], Xin-Yu Shi [1], Long-Long Zhang [1], Xiao-Zheng Fan [1] and Xiao-Jian Yang [1,*]
[1]Fourth Military Medical University, Department of Urology, Xi'an 710032, China
[2]Department of Medical Genetics, School of Basic Medical Sciences, Southern Medical University, Guangzhou 510515, China
[*]Correspondence: dryangxj@fmmu.edu.cn
[†]These authors contributed equally to this work.

Figure S1. Assessment of model confidence and structural convergence for the murine Tlr4 intracellular domain predicted by AlphaFold3.

(A-E) Predicted Aligned Error (PAE) plots for model 0 to model 4 of the Tlr4 intracellular domain (residues 660–835). The color gradient (dark green to white) represents PAE values from 0 to 30. The horizontal and vertical axes correspond to the aligned residue indices (real index).

(F) Structural superposition of DB03476 bound to Tlr4 models 0–4. Models 0, 1, 2, 3, and 4 are shown in cartoon representation colored purple, magenta, yellow, light pink, and light green, respectively. The compound DB03476 is displayed as blue sticks.

Figure S2. Docking poses of DB03476 bound to five predicted conformations (model 0–model 4) of the murine Tlr4 intracellular domain.

For each model (A–E), the left panel shows the overall binding pose, the middle panel provides a three-dimensional view of the binding environment, and the right panel presents the corresponding two-dimensional ligand–protein interaction diagram. The key residue CYS745 is highlighted and labeled in red sticks in the middle panel.

Figure S3. Molecular docking poses of the known inhibitor TAK-242 with wild-type and mutant TLR4 structures.

(A) Binding mode of TAK-242 to the Tlr4-C745A mutant (mouse). The protein is shown in light blue cartoon representation, and TAK-242 is depicted as orange sticks.

The chemical structure of TAK-242 is also provided.

(B) Binding pose and interaction details of TAK-242 with wild-type murine Tlr4. The protein is colored purple in cartoon representation.

(C) Binding pose and interaction details of TAK-242 with human TLR4. The protein is displayed in blue cartoon representation.

Figure S4. Comparative analysis of four molecular systems through all-atom molecular dynamics simulations. The systems include: apo murine Tlr4 (blue), Tlr4 in complex with DB03476 (orange), Tlr4 in complex with TAK-242 (green), and the Tlr4-C745A mutant in complex with TAK-242 (purple).

(A) Time evolution of the potential energy for each system. The three wild-type complexes (Tlr4-DB03476, Tlr4-TAK-242, and apo Tlr4) converge to comparable, stable energy levels (approximately -7.6×10^5 kJ/mol). In contrast, the Tlr4-C745A-TAK-242 system maintains a significant higher potential energy (~ -5.7×10^5 kJ/mol), indicating substantial structural instability due to the mutation.

(B) Root-mean-square deviation (RMSD) of the protein backbone over time. The Tlr4-DB03476 complex (orange) exhibits the lowest and most stable RMSD (~4 Å). The apo Tlr4 (blue) and Tlr4-TAK-242 complex (green) show moderate stability with RMSD values around 5.6-5.8 Å. The Tlr4-C745A-TAK-242 system (purple) displays markedly higher and more variable RMSD (~8 Å), confirming its pronounced conformational drift.

(C) Per-residue root-mean-square fluctuation (RMSF). Overall fluctuation patterns are similar across the wild-type and DB03476/TAK-242-bound systems. The Tlr4-C745A-TAK-242 system shows elevated fluctuations in several regions, correlating with its overall instability observed in RMSD and potential energy.

(D) Radius of gyration (Rg) over time, reflecting the overall compactness of each system. The three more stable systems maintain a consistent Rg, while the Tlr4-C745A-TAK-242 system shows a less compact and more variable profile, further evidencing its destabilized global conformation upon the loss of the key cysteine residue.

Figure S5. Structural superposition and final binding poses from 100 ns molecular dynamics simulations for three ligand-Tlr4 complexes.

(A) Overlay of the Tlr4-DB03476 complex before (protein in light purple cartoon, DB03476 in light blue sticks) and after (protein in deep purple cartoon, DB03476 in deep blue sticks) the 100-ns simulation, alongside the final stable binding pose.

(B) Overlay of the Tlr4-TAK-242 complex before (TAK-242 in orange sticks) and after (TAK-242 in yellow sticks) the 100 ns simulation, with the corresponding final binding pose. The protein is shown in cartoon representation.

(C) Overlay of the Tlr4-C745A-TAK-242 complex before (protein in light blue cartoon) and after (protein in gold cartoon) the 100 ns simulation, together with its final conformation. TAK-242 is depicted in stick representation.

Figure S6. DB03476 ameliorates LPS-induced macrophages polarization

(A) Immunofluorescence staining for TLR4 in drug-treated macrophages. Bars = 40 μm.

(B) The protein level of TLR4 in drug-treated macrophages was detected by Western blotting.

(C-D) Immunofluorescence staining for iNOS, and Arg1 in drug-treated macrophages. The sample size was n=3 per group. Data are presented as mean ± SEM. ***P<0.01, ***P<0.001, ****P<0.0001.*

Figure S7. H&E staining of major organs (heart, liver, spleen, lung) collected from C57BL/6 mice 7 days after treatment with Saline or DB03476.

Figure S8. Effects of DB03476 on the expression of macrophage polarization markers CD86/CD206 and other TLR family members in AKI model kidneys

(A-B)Western blotting images of CD86 and CD206 protein expression in renal tissues from IRI + Saline and IRI + DB03476 groups. (C-E) Western blotting images of TLR2 (C), TLR7 (D), and TLR8 (E) protein expression in renal tissues. Data are

presented as mean $\pm$ SEM (n=3 per group). $^{**}P<0.01$, $^{***}P<0.001$, $^{****}P<0.0001$.
