# Supplementary figures and images for: A Novel TLR4 Inhibitor DB03476 Rescued Renal Inflammation in Acute Kidney Injury Model
Source: Int J Mol Sci. 2025 Dec 31;27(1):454. doi: 10.3390/ijms27010454 (PMC12787066; doi:10.3390/ijms27010454)

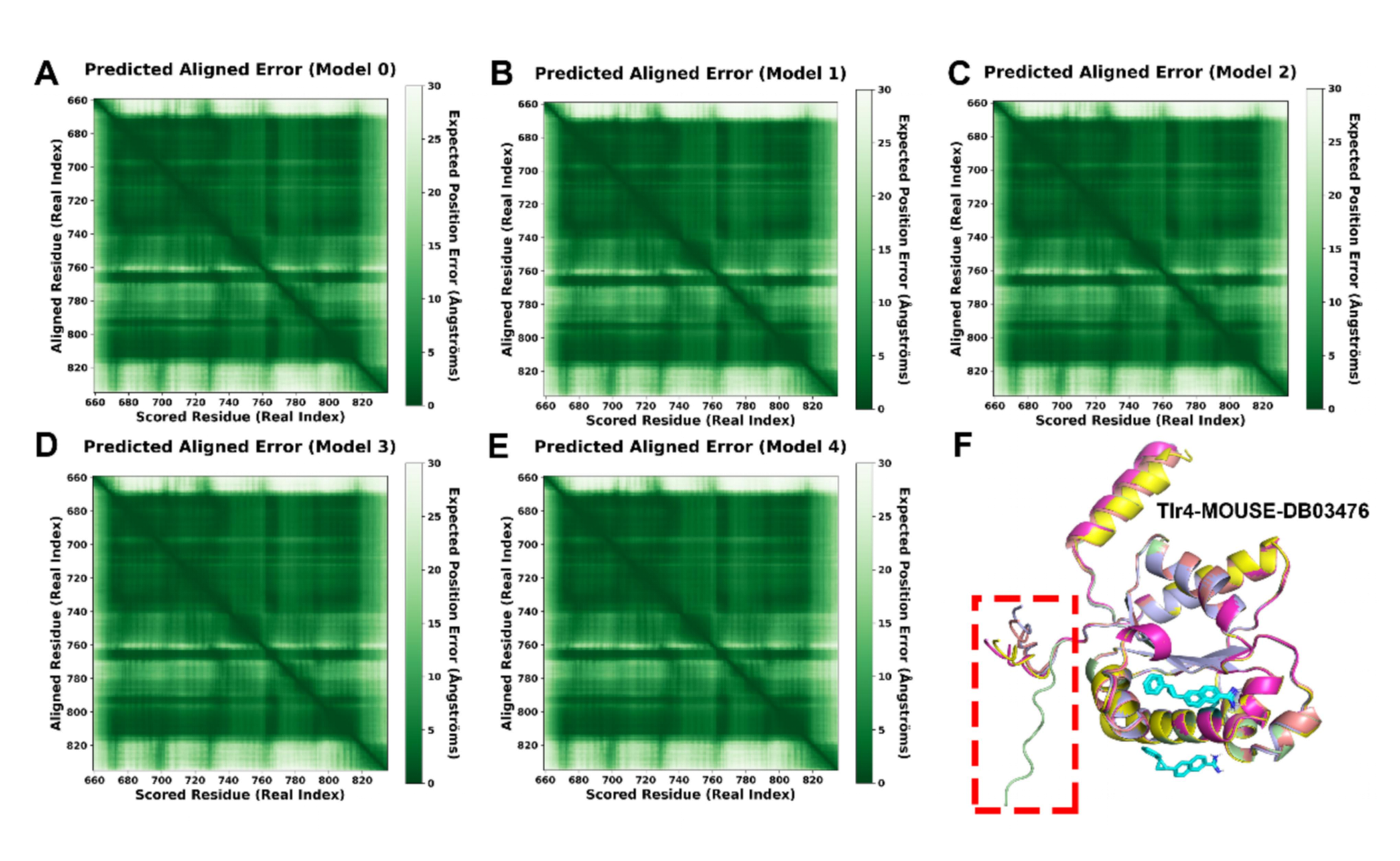

Supplement: Supplementary file 1 [file ijms-27-00454-s001.zip › Supplementary Materials1.tif]

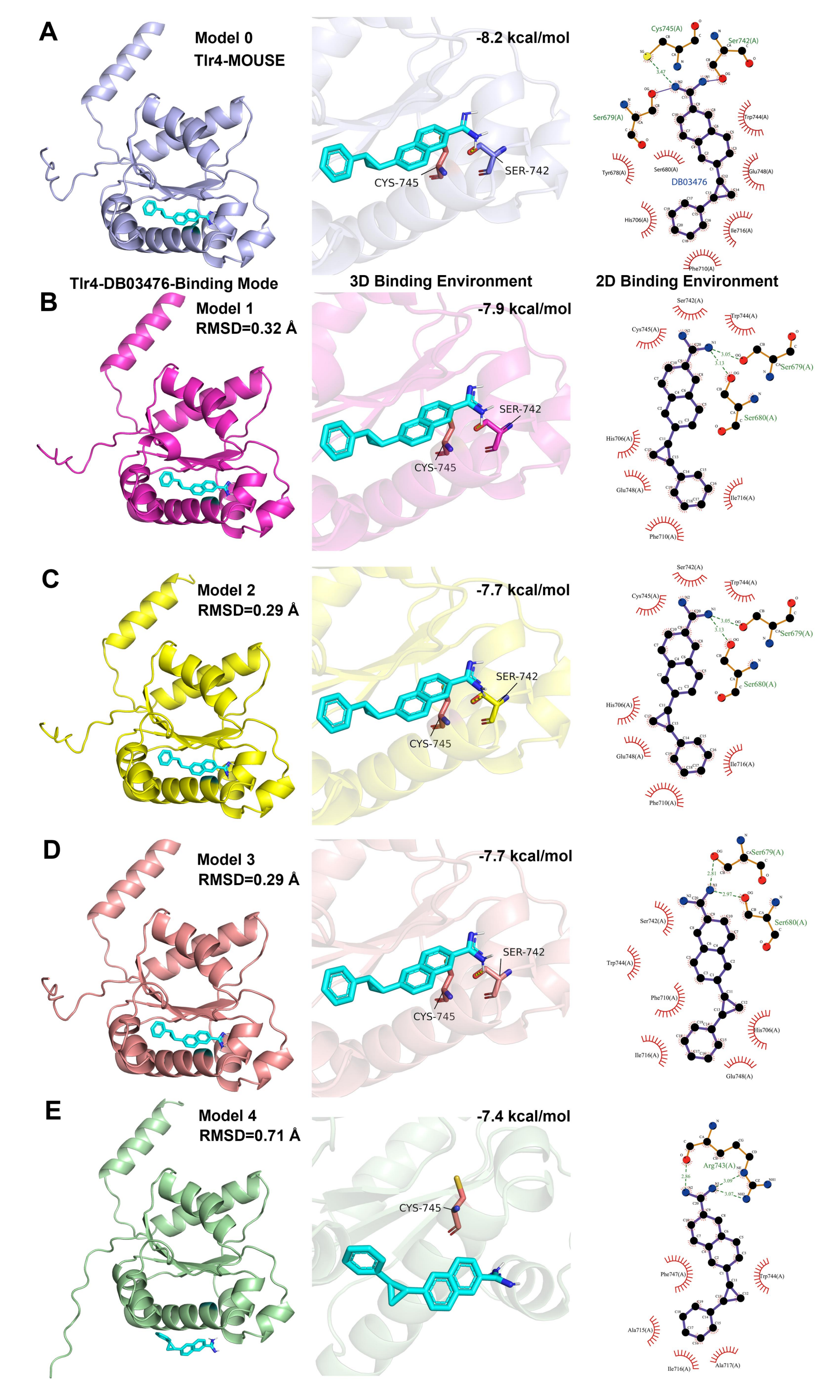

Supplement: Supplementary file 1 [file ijms-27-00454-s001.zip › Supplementary Materials2.tif]

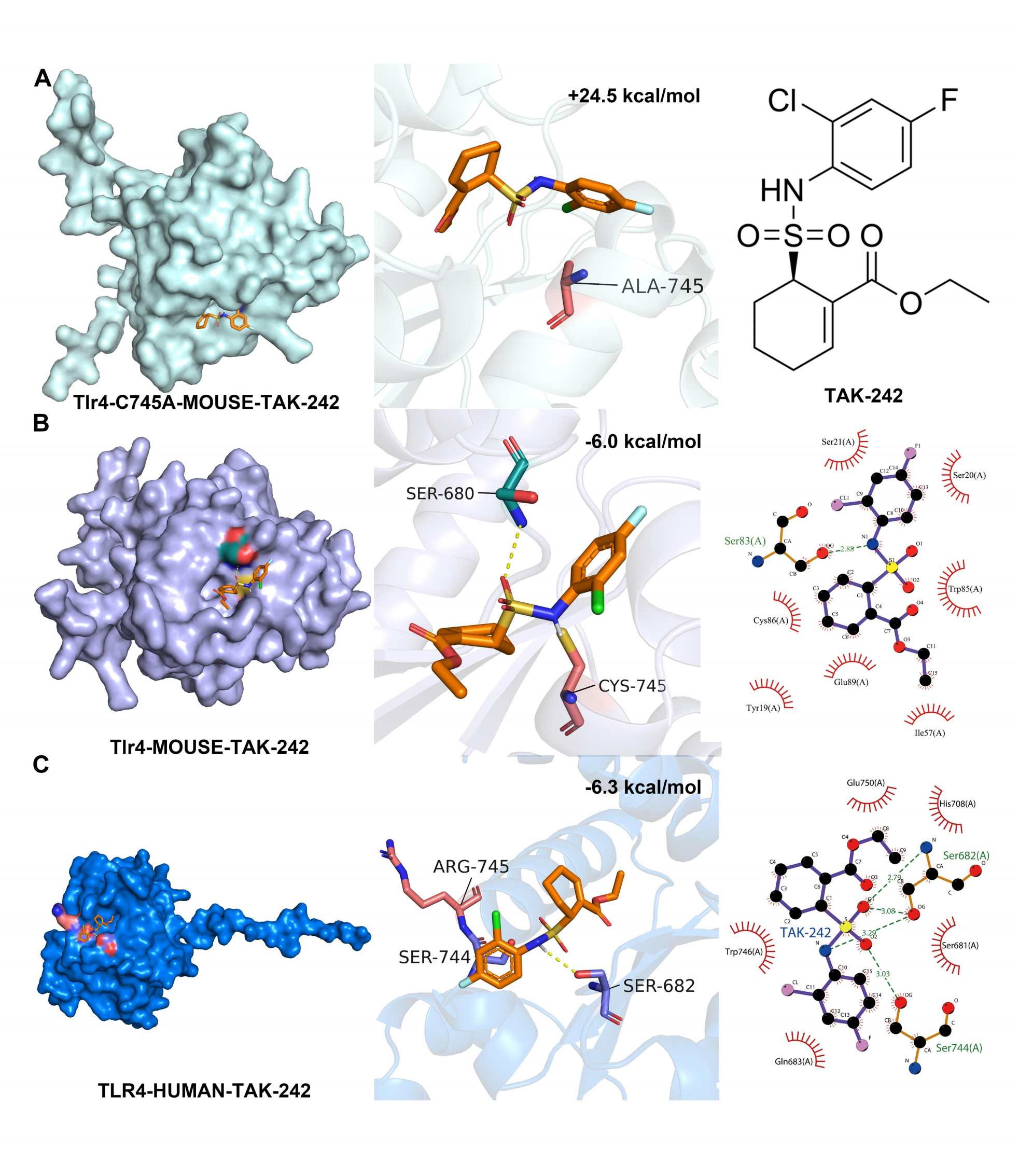

Supplement: Supplementary file 1 [file ijms-27-00454-s001.zip › Supplementary Materials3.tif]

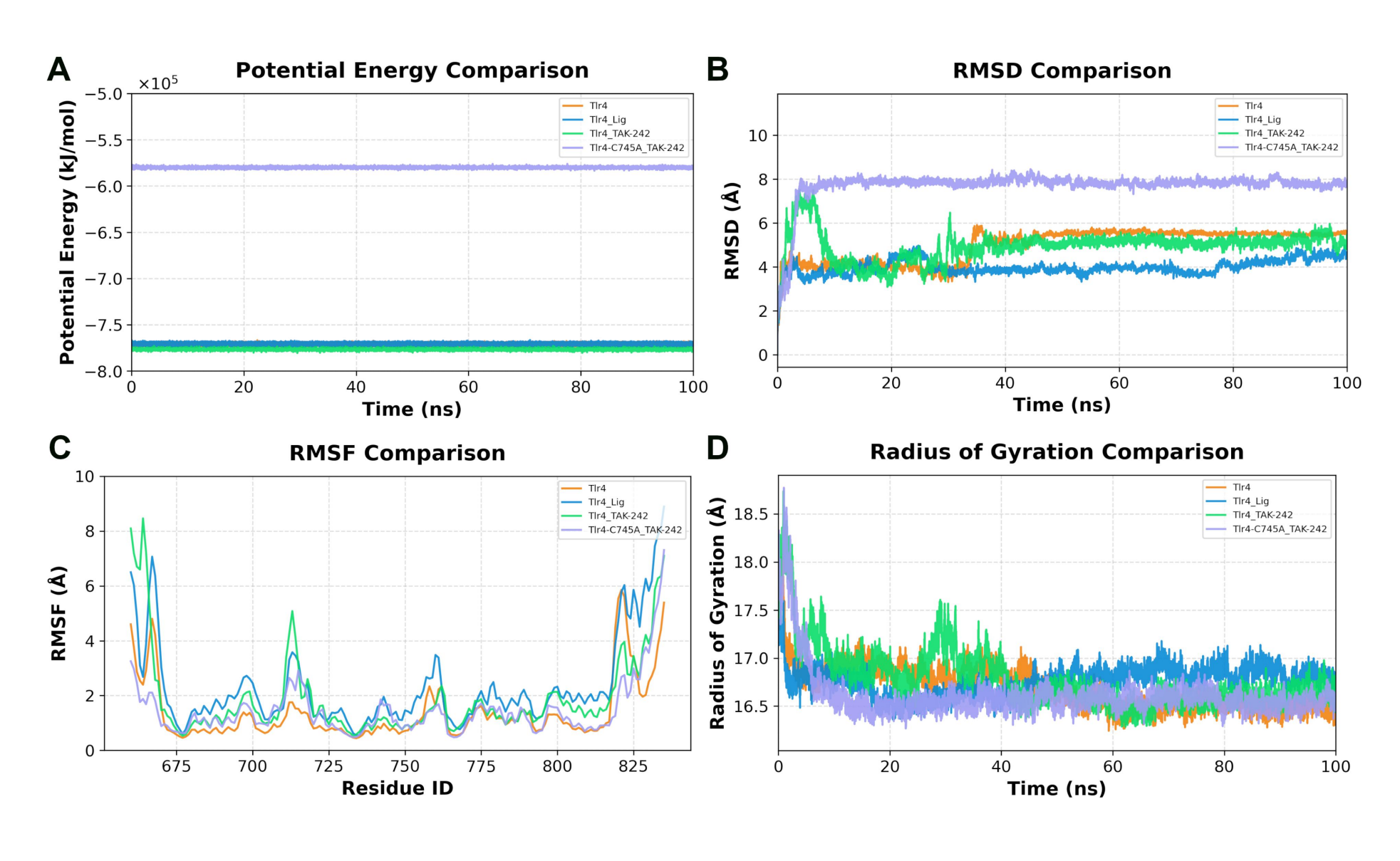

Supplement: Supplementary file 1 [file ijms-27-00454-s001.zip › Supplementary Materials4.tif]

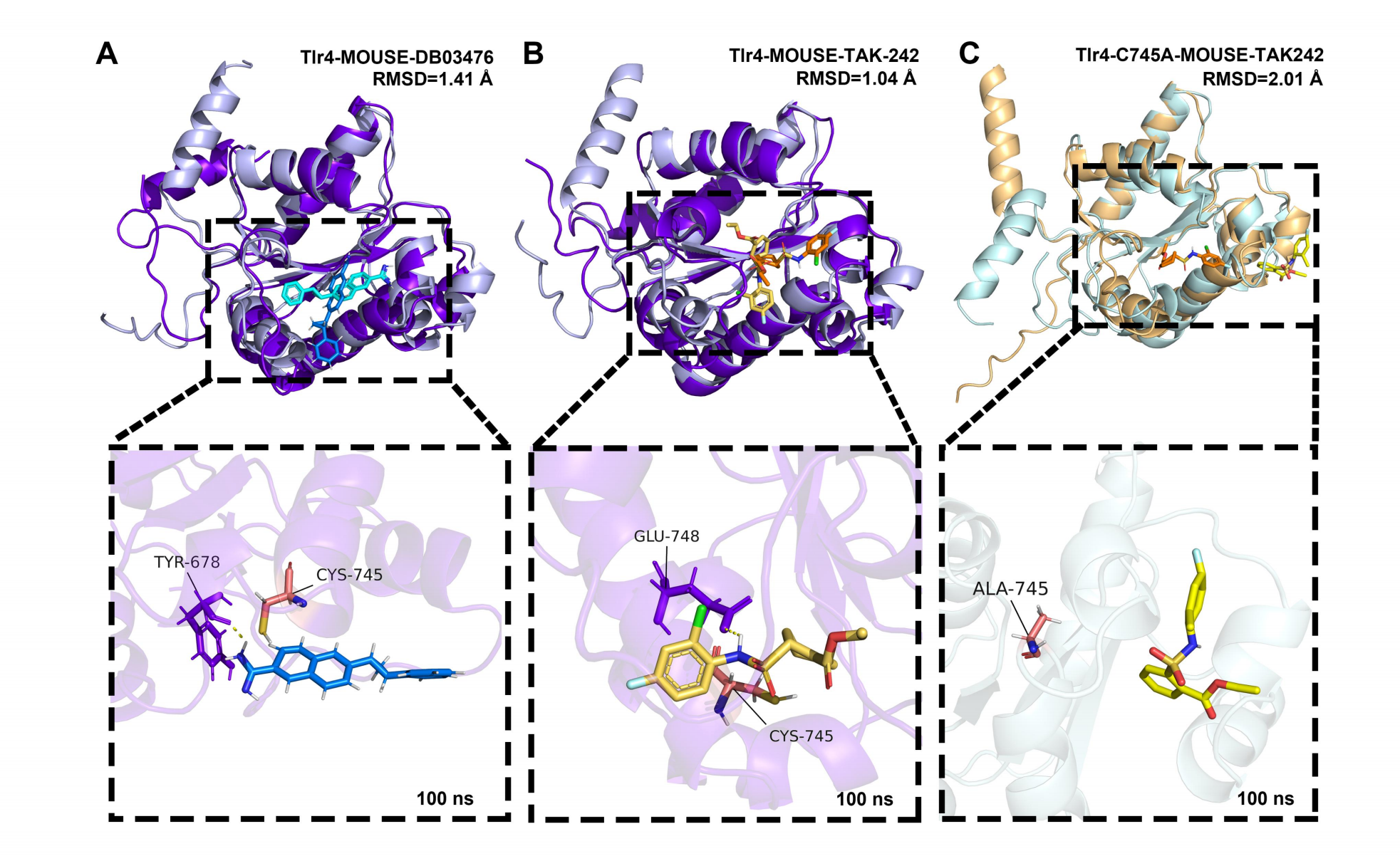

Supplement: Supplementary file 1 [file ijms-27-00454-s001.zip › Supplementary Materials5.tif]

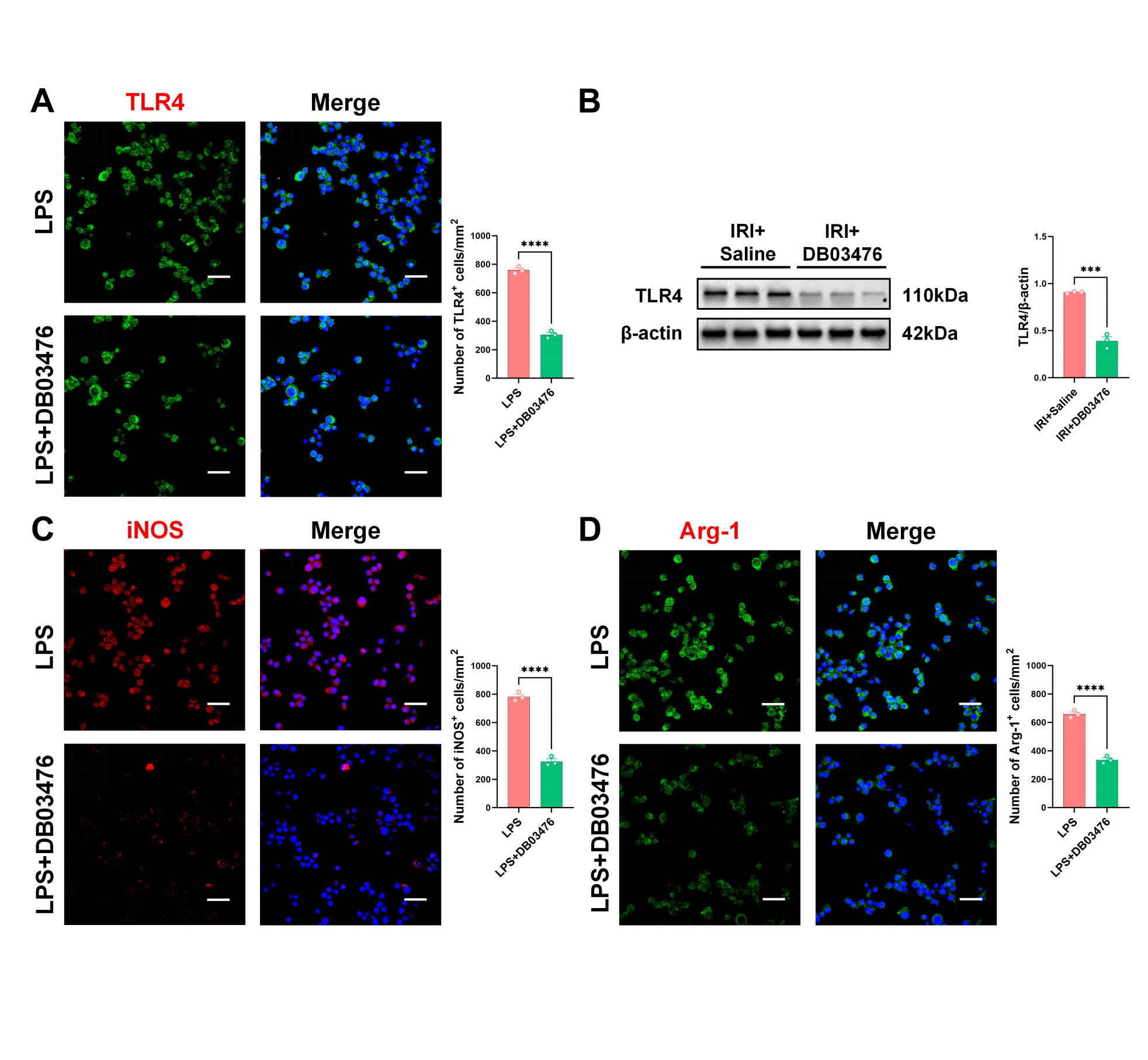

Supplement: Supplementary file 1 [file ijms-27-00454-s001.zip › Supplementary Materials6.tif]

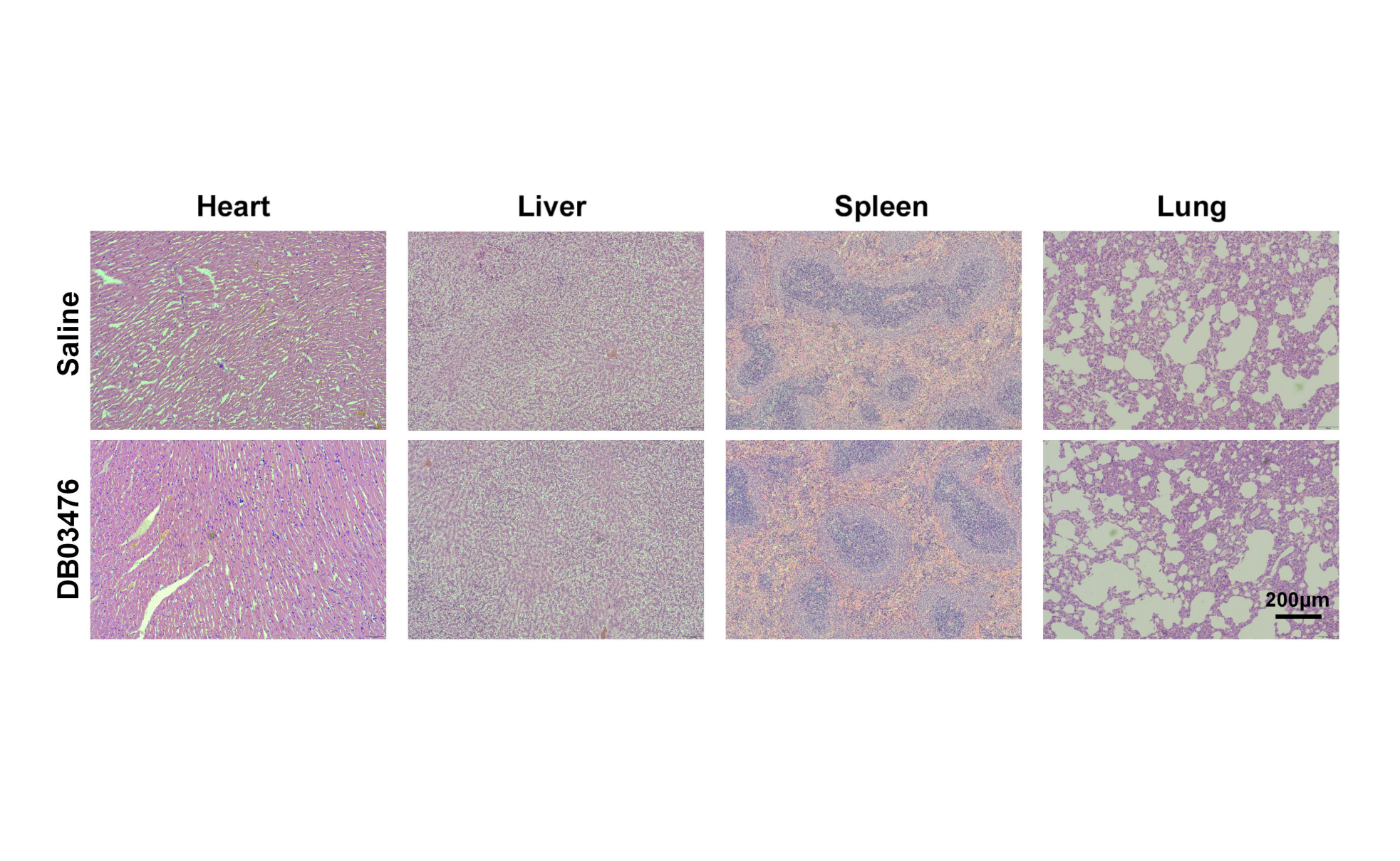

Supplement: Supplementary file 1 [file ijms-27-00454-s001.zip › Supplementary Materials7.tif]

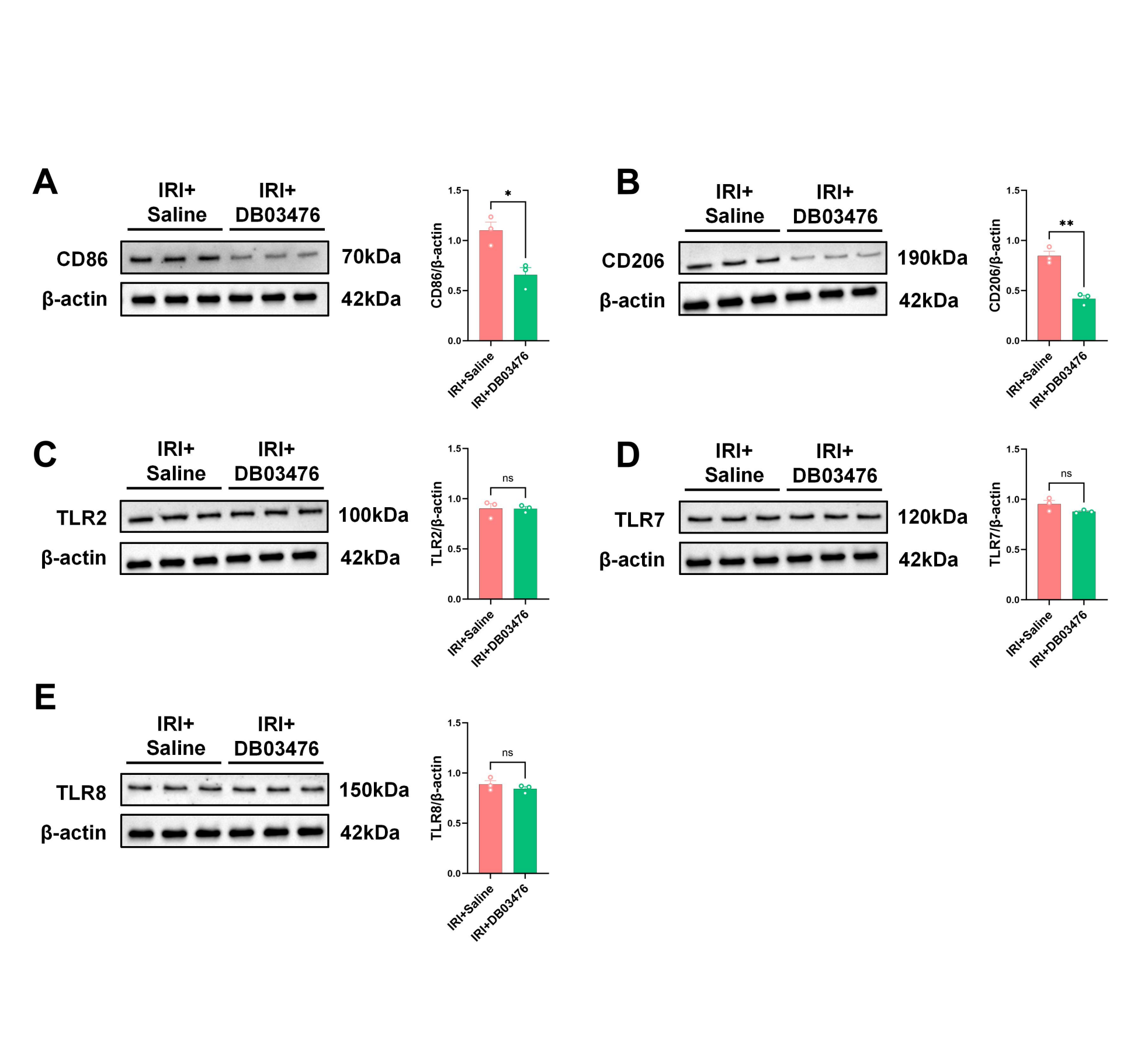

Supplement: Supplementary file 1 [file ijms-27-00454-s001.zip › Supplementary Materials8.tif]
